# Evaluation of elastin fibres in young and aged eyelids and abdominal skin using computational 3D structural analysis

**DOI:** 10.1002/ski2.58

**Published:** 2021-06-22

**Authors:** T. Tohgasaki, S. Kondo, S. Nishizawa, S. Ishiwatari, T. Sakurai, S. Ishikawa, A. Takeda

**Affiliations:** ^1^ Fancl Research Institute Fancl Corporation Yokohama Kanagawa Japan; ^2^ Department of Plastic and Aesthetic Surgery Kitasato University School of Medicine Sagamihara Japan

## Abstract

**Background:**

Aging‐related degeneration of elastic fibres causes skin wrinkles and loss of elasticity. A correlation has been reported between dermal elastic fibre degradation and wrinkles. However, the mechanism of wrinkle formation is complex and unclear. To establish methods for treating wrinkles, it is necessary to understand the aging‐related morphological alterations underlying elastin fibre degradation or disappearance.

**Objectives:**

To image and analyse aging‐related three‐dimensional (3D) morphological alterations of elastic fibres in the eyelid and abdominal skin.

**Methods:**

Excised human eyelid and abdominal skin tissues were examined. The structure of elastic fibres in the skin tissues was examined via nuclear, tropoelastin and fibrillin‐1 immunostaining. Then, 3D imaging was performed using a confocal laser microscope and tissue decolourization technology. Images were analysed using a computational method.

**Results:**

The decolourization technology made it possible to image elastin fibres in 3D, and we devised a method for analyzing the elastin fibre structure using computational methods. It was quantitatively shown that the eyelid skin has a more complex fibrous structure than the abdomen, and the fibres became curved, shortened and thickened with age.

**Conclusions:**

We provide a novel 3D analysis method for elastin fibres and report age‐related alterations in elastin fibre structure in the human eyelid and abdominal skin. This method contributes to the understanding of elastin fibre degeneration in more detail than conventional methods. Applying this 3D analysis method to skin tissues will contribute to a better understanding of age‐related changes in fibres and to the development of novel wrinkle treatments.

## Graphical abstract

Aging‐related degeneration of elastic fibers causes skin wrinkles and loss of elasticity. Here, we applied three‐dimensional imaging and a novel analysis method using immunostaining with decolorization technology and computational method to abdominal and eyelid skin tissues to evaluate aging‐related alterations in elastic fiber. We showed numerical differences in elastin fibers in the eyelids and abdominal skin excised from young and elderly patients. Hence, our analysis method contributes to the understanding of elastin fiber structure and further development of wrinkle treatments.

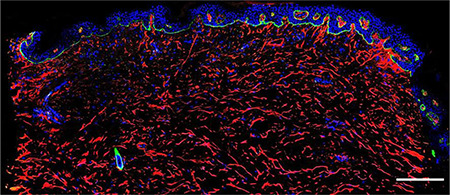

1

**What is already known about this topic?**

- Dermal elastic fibre degradation and wrinkles correlate.
- The mechanism of wrinkle formation is complex and unclear.
- It is necessary to understand the morphological alterations that occur with aging.

**What does this study add?**

- We provide a novel 3D analysis method for elastin fibres.
- This method contributes to the understanding of elastin fibre degeneration in more detail than conventional methods.

**What are the clinical implications of this work?**

- The 3D analysis method described here will contribute to a better understanding of age‐related changes in elastin fibres and to the development of novel wrinkle treatments.

## 2 INTRODUCTION

Skin wrinkles are visible symptoms of aging, affecting physical appearance, mental health and quality of life.^1^ Therefore, effective wrinkle treatment is desirable. However, further studies are needed to establish methods to effectively treat wrinkles.

Elastic fibres, components of the dermal extracellular matrix, provide elasticity and structural integrity to the skin.^2^, ^3^ Elastic fibres can be divided into three types: elastic, elaunin and oxytalan fibres.^4^ In the reticular dermis, elastic fibres extend parallel to the skin surface and interact with elaunin fibres. Elaunin fibres are slightly thicker and extend to the papillary layer base from the reticular dermis. Oxytalan fibres extend from elaunin fibres to the dermal‐epidermal junction and their terminals contain a large proportion of fibrillin. In particular, the papillary dermis is rich in oxytalan fibres and exhibits candelabra‐like structures.^5^, ^6^ These fibres are associated with skin resilience and skin surface texture and help resist aging‐related changes in the skin.^7^

Several molecules related to the formation of elastic fibres have also been reported. The formation of elastic fibres includes tropoelastin deposition (the soluble precursor of mature elastin) on a preformed scaffold of fibrillin‐rich microfibrils. In addition, the cross‐linking enzyme links these intermolecular structures to form insoluble fibres.^8^ Various proteins (e.g., lysyl oxidase, fibulin‐5 and elastin microfibrillar interface protein) are involved in this process.^8^ In contrast, aging‐related degeneration of elastic fibres causes skin wrinkles and loss of elasticity.^9^ Studies using skin sections from old and young individuals have revealed a correlation between dermal elastic fibre degradation and wrinkles.^10^ Both intrinsic and extrinsic factors induce degeneration of elastic fibres. The dermis of photoprotected aged skin has fewer fibroblasts and degenerated elastic fibres than the dermis of young skin.^11^ Exposure to ultraviolet rays further accelerates dermal aging.^12^, ^13^ Ultraviolet irradiation induces elastosis by increasing the expression of elastin. Some elastin proteases degrade elastic fibres and deposit fragments in the dermis.^14^, ^15^ Thus, photoaged skin shows an abnormal accumulation of elastic fibre fragments.^14^ Besides, elastin‐degrading enzymes, matrix metalloproteinase‐2, ‐3, ‐7, ‐9 and ‐12, as well as neutrophil serine proteases, cathepsin G and human leukocyte elastase, are released during ischemia, inflammation and chronic mechanical irritation.^14^, ^15^, ^16^, ^17^, ^18^, ^19^, ^20^, ^21^ These enzymes cause the loss of dermal elastic fibres in the acquired form of cutis laxa.^16^ These alterations in the fibrous structure of the skin with age are associated with wrinkle formation and skin elasticity.^22^, ^23^

The mechanism of wrinkle formation is complex and unclear. To establish methods for treating wrinkles, it is necessary to understand the morphological alterations underlying degradation or disappearance with aging. To date, most studies on wrinkle structures have been performed using histological techniques involving histochemistry, immunohistochemistry and electron microscopy. However, two‐dimensional (2D) observations are limited, thereby impeding the understanding of the elastic fibre structure that extends deep into the skin better. Recently, three‐dimensional (3D) imaging combined with decolourization and fluorescent immunostaining has been used to facilitate the visualization of several tissues, organs and whole mice.^24^, ^25^, ^26^, ^27^, ^28^, ^29^ In this study, we aimed to apply the decolourization technique to excised human skin tissue and to observe the 3D structure of elastin fibres. Moreover, to evaluate the elastin fibre structure objectively and quantitatively, we established a computational 3D structural analysis method for 3D imaging. We investigated the age‐related alterations of elastin fibres in the human abdominal skin tissue as the unexposed area and the eyelid skin tissue as the exposed area, using the computational 3D structural analysis method.

## 3 MATERIALS AND METHODS

### 3.1 Excised skin tissue

Abdominal and eyelid skin were used to evaluate the age‐ and site‐dependent differences in fibrous structures of the skin. Human abdominal skin tissues from six Caucasian women (aged, 23, 24, 27, 61, 61 and 64 years) fixed with 4% paraformaldehyde (PFA) in phosphate‐buffered saline (PBS) were purchased from KAC Co., Ltd. Human eyelid skin tissues from six Japanese women (aged, 11, 12, 20, 72, 80 and 87 years) were provided by Kitasato University School of Medicine and fixed with 4% PFA in PBS. To evaluate the differences between young and aged individuals, we divided the samples as young (from donors in their 10s or 20s) and aged (from donors in their 60s). The study protocol conformed to the ethical guidelines of Kitasato University's ethics committee (B16‐285) and was conducted according to the principles of the Declaration of Helsinki. Informed consent was obtained from each patient before enrolment in the study.

### 3.2 Fluorescence immunostaining and decolourization

Each fixed skin tissue was sliced into 1‐mm sections in the epidermis‐dermis orientation. The skin sections were washed with 0.05% Tween 20 in PBS and blocked with StartingBlock blocking buffer (Thermo Fisher Scientific). Thereafter, they were labelled with anti‐tropoelastin mouse monoclonal antibody (Merck Millipore) and anti‐fibrillin‐1 rabbit polyclonal antibody (Elastin Products Company, Inc.) diluted in StartingBlock blocking buffer at 1:500. After washing, the sections were stained with goat anti‐mouse immunoglobulin G (IgG) H&L Alexa Fluor^®^ 488 and goat anti‐rabbit IgG H&L Alexa Fluor^®^ 647 (Thermo Fisher Scientific) at 1:1000 dilution. Nuclear staining was performed using 4',6‐diamidino‐2‐phenylindole (DAPI) (DOJINDO Lab) at 1:5000 dilution. After washing, the sections were subsequently decolourized with Rapiclear 1.49 (SunJin Lab Co.).

### 3.3 3D imaging and computational 3D structural analysis

**FIGURE 1:**
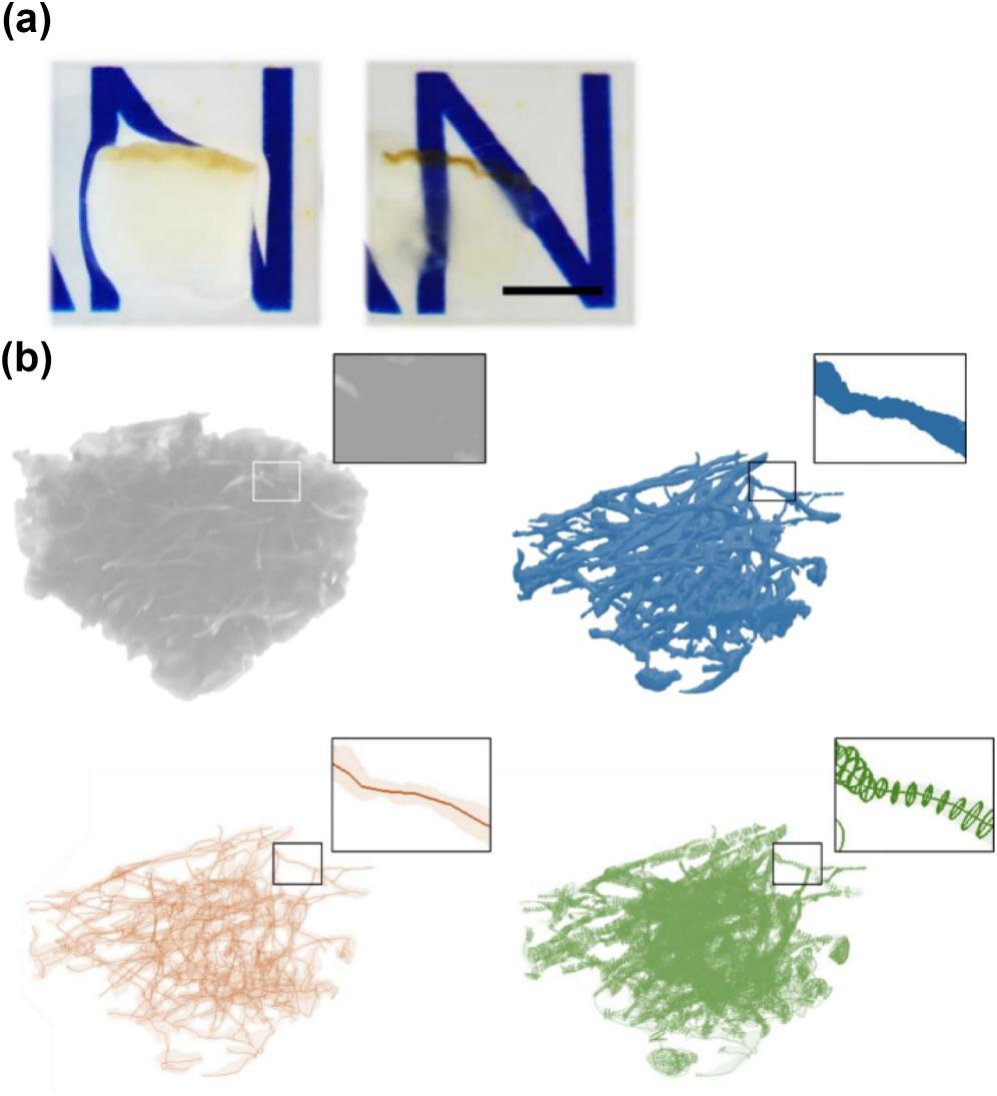
Decolourization and computational three‐dimensional (3D) structural analysis of elastin fibres. Each skin tissue was decolourized by applying Rapiclear 1.49 after immunostaining. Skin tissue before (left) and after (right) decolourization. Scale bar = 3 mm. (a) Processing of 3D images for structural analysis. (b) Images of elastin fibres captured with a confocal microscope were reconstructed in 3D (grey) and processed in three steps. Post‐binarization image (blue), extracted fibrous centreline (red) and cross sections of the fibres (green). Each square frame presents a magnified view of the respective image

After decolourization (Figure 1a), nuclear, tropoelastin and fibrillin‐1 in each skin section were observed in the direction of epidermis‐dermis using a confocal laser scanning microscope (Olympus FV‐1000) with a 60× silicon lens. Using the tiling function attached to the microscope, cross‐sectional images (*X*:*Y* = 450:450 μm) were acquired continuously every 0.8 μm (*Z*‐stacks) for a total depth of 80–150 μm. The captured images of elastin fibres were processed for binarization, and the volume ratio of the fibre to the whole visual field and the surface area of the fibre were measured. The skeletonization of binarized fibre was performed by extracting the centre of gravity and fitting the point, and the number of fibre branches, fibre length, thickness and degree of curvature were measured. The degree of curvature was calculated by averaging the second derivative value at each 1 μm point at the gravity centre of the fibre. Figure 1b shows the images processed using each method. These structural analyses were performed using the 3D image data processing software, Simpleware (JSOL).

### 3.4 Statistical analysis

Data from the experiments are expressed as the mean ± standard deviation for each test group. Differences between groups were evaluated using Student's *t*‐test. All statistical analyses were performed using the Bell Curve for Excel version 7.0 (Social Survey Research Information Co., Ltd). Results were considered significant at *p* < 0.05 (*p < 0.05, **p < 0.01 and ***p < 0.005).

## 4 RESULTS

### 4.1 The application of tissue decolourization technology‐enabled 3D imaging of elastin fibres in the skin tissue

**FIGURE 2:**
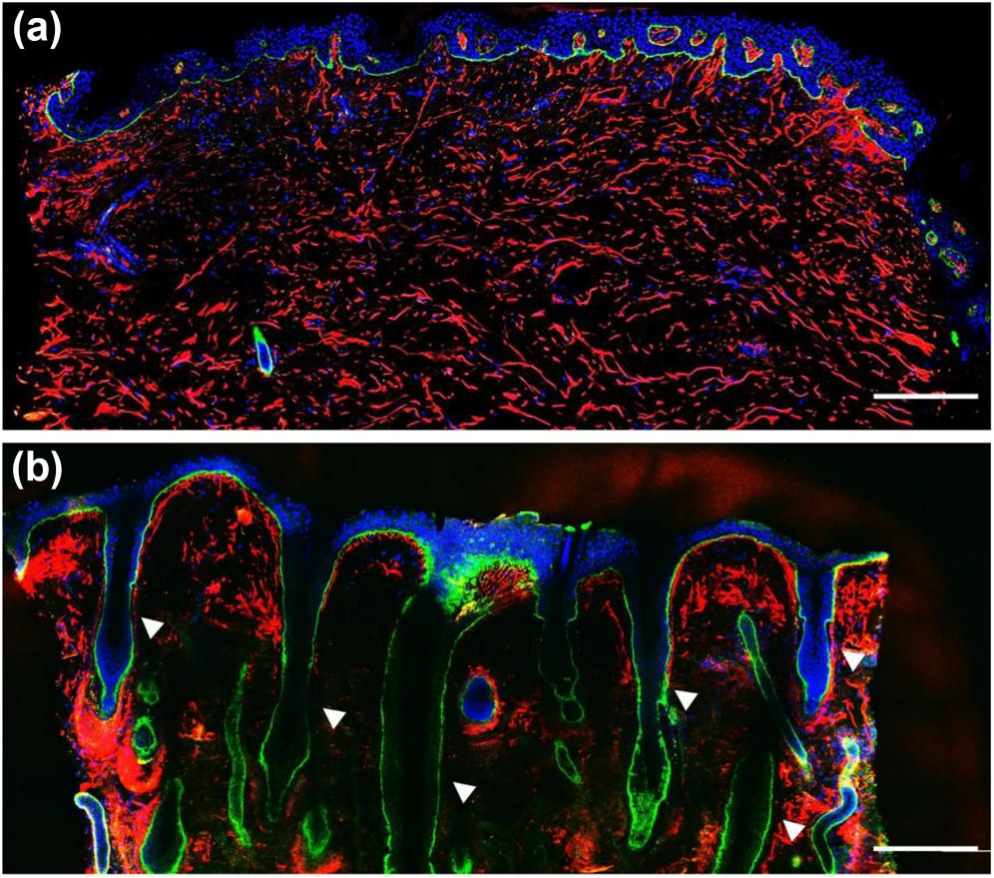
Whole section images of abdomen and eyelid skin tissues. Whole section images of abdominal skin from a 23‐year‐old donor (a) and eyelid skin from a 20‐year‐old donor (b). Each image was stitched by tiling using an electric stage. Blue: DAPI, green: collagen‐7 and red: tropoelastin. Scale bar = 250 μm. The white arrow indicates the appendages. DAPI, 4',6‐diamidino‐2‐phenylindole

To evaluate the 3D structure of elastin fibres in the dermis, abdominal and eyelid skin tissues were examined with nuclear, tropoelastin and fibrillin‐1 staining and decolourized. Thereafter, 3D images of each structure in the skin tissue were continuously obtained in the epidermis‐dermis orientation. As a result, although this imaging method allowed us to observe up to a depth of 150 μm, the fluorescence signal was reduced at depths greater than 100 μm. The structure that appeared as a dot in a 2D image was linear in the 3D rebuilt images (Movie [Link], [Link]). This difference in appearance indicated that 3D imaging enabled an accurate understanding of the structure of elastin fibres in the dermis. Moreover, to understand the differences in the number of appendages, such as hair follicles and sweat glands in the abdomen and eyelids, we captured whole skin tissue images. Figure 2 shows the whole image of a section of the abdomen of a 23‐ and a 20‐year‐old‐woman. Appendages were often observed in the eyelid but rarely in the abdominal skin. Therefore, we evaluated the 3D structure of elastin fibres in each skin tissue, focusing on the region under the basement membrane and without any appendages, to determine the effects of aging and the differences depending on the site. Moreover, when we performed the computational analysis, we used 3D images up to a depth of 100 μm.

### 4.2 Comparison of elastin fibre structures in the abdomen and eyelid skin by observing 3D images

**FIGURE 3:**
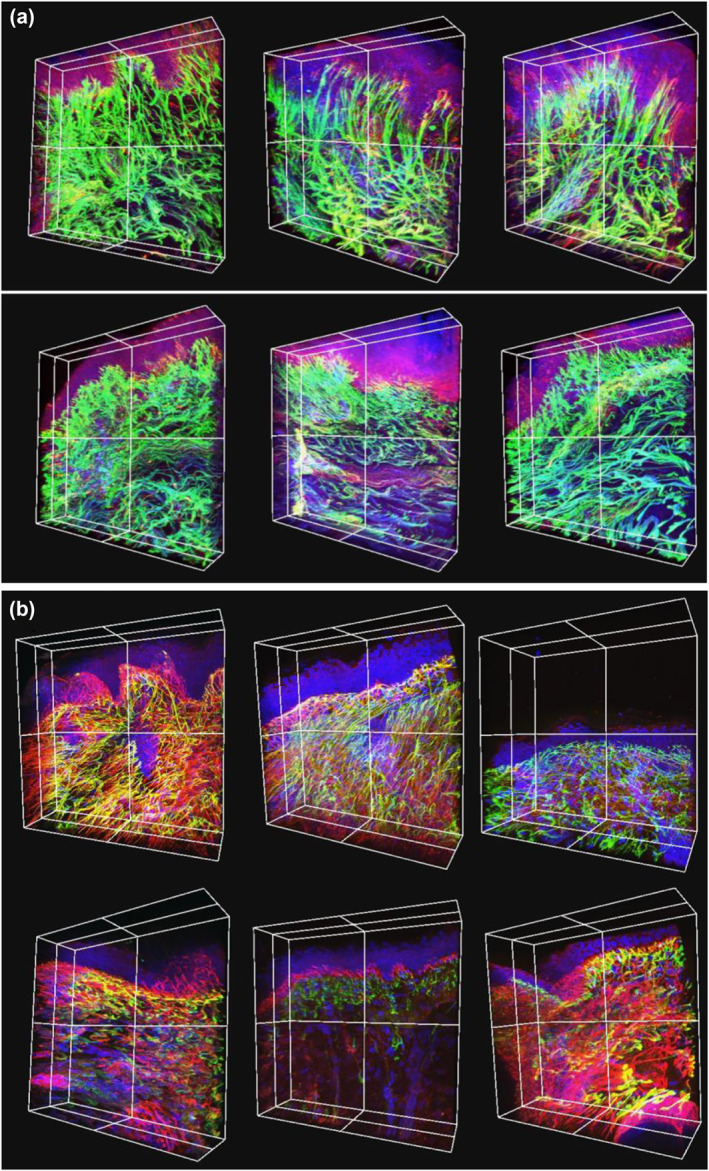
Fluorescence images of abdominal and eyelid skin tissues. Three‐dimensional images of elastin fibres in the human abdomen (a) and eyelid (b) skin samples. Abdominal skin samples from young (upper: 23‐, 24‐ and 27‐year‐old, from left) and aged (lower: 61‐, 61‐ and 64‐year‐old, from left) donors. Eyelid skin samples from young (upper: 11‐, 12‐ and 20‐year‐old, from left) and aged (lower: 72‐, 80‐ and 87‐year‐old) donors. Blue: DAPI, green: tropoelastin and red: fibrillin‐1. DAPI, 4',6‐diamidino‐2‐phenylindole

To investigate the age‐related alterations of elastic fibres in the abdominal and eyelid human skin, abdominal skin and eyelids excised from six females were observed. The typical images are shown in Figure 3. Tropoelastin and fibrillin‐1 in the abdominal skin dermis were generally co‐localized in a fibrous shape, but oxytalan fibre tips were rich in fibrillin‐1. Oxytalan fibres showed a candelabra‐like structure extending to the dermal‐epidermal junction from the arched fibres, parallel to the basement membrane. These structures were clearly and frequently observed, especially in the papillary layer of the young skin. In a slightly deeper region of the dermis, elaunin fibres were observed, extending parallel to the epidermal basement membrane. The fibrous structure in aged abdominal skin was shorter and more curved than in the young abdominal skin (Figure 3a). In contrast, tropoelastin and fibrillin‐1 were localized in the elastin fibre in the eyelid skin, similar to the abdominal skin. However, the co‐localization of tropoelastin and fibrillin‐1 was lower in the eyelid skin than in the abdominal skin. Moreover, the eyelid skin oxytalan fibres were observed as a complicated network under the base membrane but hardly observed as a candelabra cascade; they were short, small and few in numbers. In a slightly deeper region, elaunin fibres extended in multiple directions. In the aged eyelid skin, elastin fibres were shorter, curved and shrunken, similar to the aged abdominal skin. In addition, oxytalan fibres were rarely observed in the aged eyelid skin (Figure 3b).

### 4.3 Elastic fibre structure was shorter, thicker and curved in aged samples

**FIGURE 4:**
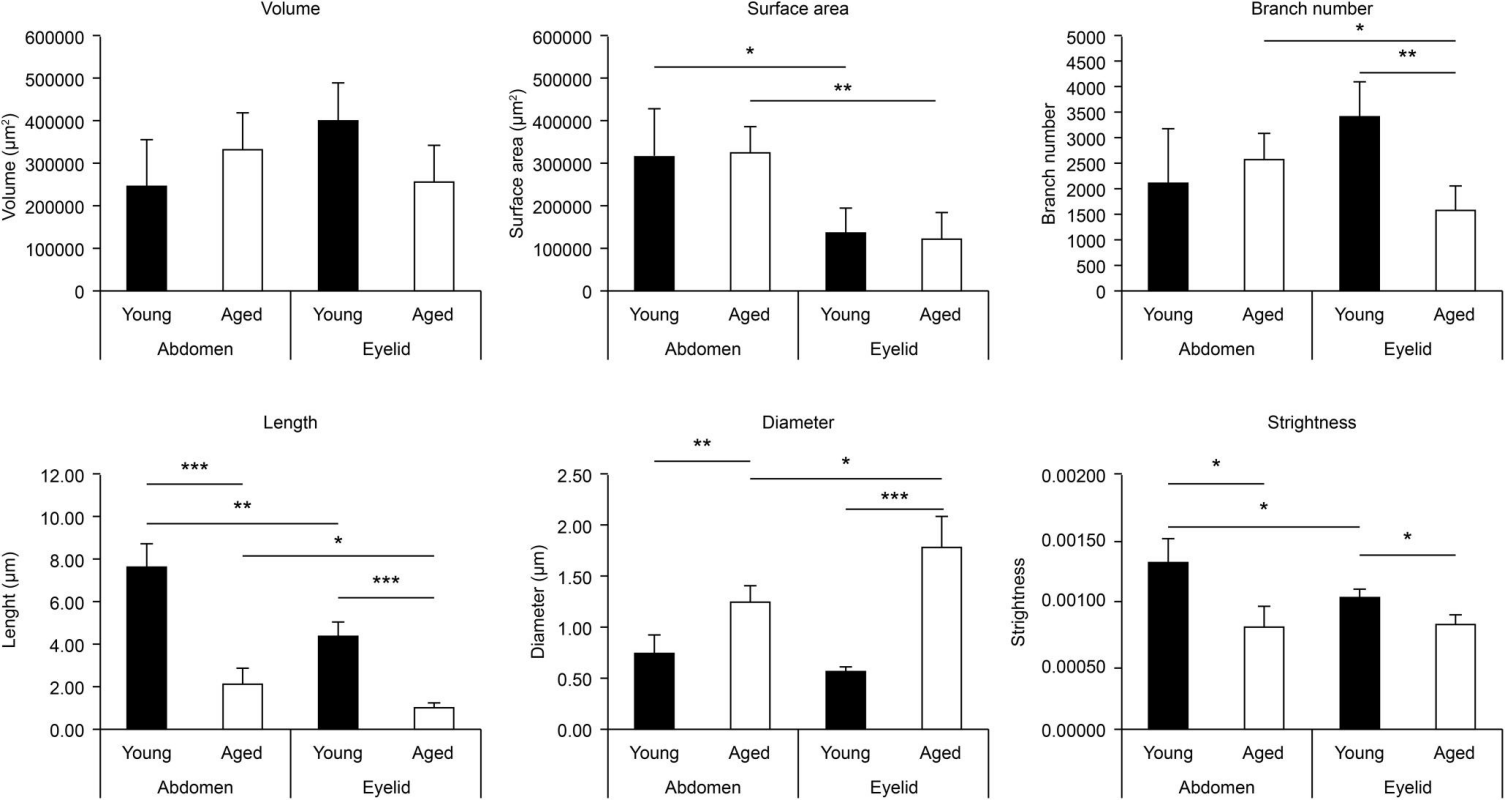
Comparison of elastin fibre characteristics using computational three‐dimensional (3D) structural analysis. Calculated values of 3D structural analysis for abdominal skin samples from young (23 to 27‐year‐old; *n* = 3) and aged (61 to 64‐year‐old; *n* = 3) donors and eyelid skin samples from young (10 to 20‐year‐old; *n* = 3) and aged (72 to 87‐year‐old; *n* = 3) donors. Data were analysed using the Mann–Whitney *U* test. *p < 0.05, **p < 0.01, ***p < 0.005

**FIGURE 5:**
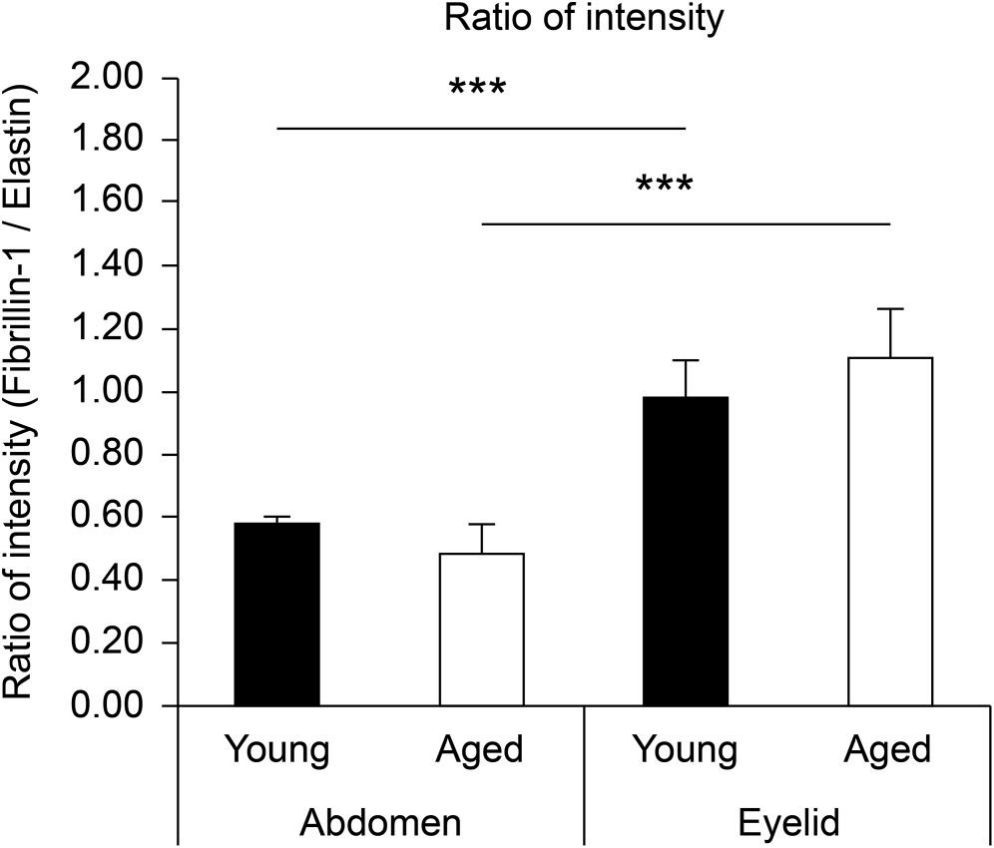
Comparison of the fluorescence intensity of fibrillin‐1 and elastin. Ratio of the fluorescence intensity of fibrillin‐1 and tropoelastin for abdominal skin samples from young (23 to 27‐year‐old; *n* = 3) and aged (61 to 64‐year‐old; *n* = 3) donors and eyelid skin samples from young (11 to 20‐year‐old; *n* = 3) and aged (72 to 87‐year‐old; *n* = 3) donors. Data were analysed using the Mann–Whitney *U* test. Error bars represent standard deviation. ***p < 0.005

To evaluate the elastin fibre structure objectively and quantitatively, we applied the 3D image data processing software, Simpleware, to the 3D images of tropoelastin staining. Thereafter, the volume, surface area, number of branches, length, diameter and elastin fibre curvature were calculated. The computational analysis revealed that elastin fibres in the aged abdominal skin were 28% longer, 165% thicker and 165% more curved than in the young abdominal skin. In contrast, elastin fibre in the eyelid skin was 25% longer, 309% thicker and 125% more curved than in the young eyelid skin. In addition, the branch number of the aged eyelid skin was decreased by 45% compared to that of the young skin, while there were no alterations in that of the abdominal skin. There were no differences in the volume and surface area between young and aged skins. Fibres in the abdominal skin were significantly larger than in the eyelid skin. Besides, fibre length in the aged eyelid skin was shorter than that in the aged abdomen skin, but there was no significant difference in the young skin (Figure 4). These data suggested that elastic fibres became shorter, thicker and more curved with age, and these alterations were more dramatic in the eyelid skin. We calculated the ratio of elastin and fibrillin‐1 fluorescence signal intensity (fibrillin‐1/tropoelastin) using Image J software. Elastin fibres in eyelid skin were found to be richer in fibrillin‐1 than in the abdominal skin (Figure 5).

## 5 DISCUSSION

In this study, to better understand the elastin fibre structure, we applied tissue decolourization technology to extracted skin tissues and established a novel computational 3D structural analysis for the immunofluorescent 3D images. We applied this method to the 3D images of elastin fibres in young and aged, exposed (eyelid) and unexposed (abdomen), skin tissues to evaluate age‐related alterations in elastic fibres of the skin.

Rapiclear 1.49, a decolourization reagent, made it possible to observe the skin tissue up to a depth of 150 μm. By performing 3D observations, we confirmed a linear structure, while this structure looked different in 2D observations. 3D imaging enabled us to understand the structure of elastin fibres that were erroneously recognized using 2D observation. However, these 3D images provided a large amount of information compared to 2D images, and it was difficult to evaluate them correctly. Therefore, computational 3D structural analysis was used to objectively and quantitatively evaluate 3D images. We then applied a 3D image data processing software, Simpleware, which is used to analyse x‐ray images, to evaluate elastin fibres. Then, elastin fibre structural parameters, such as volume, surface area, number of branches, length, diameter, and curvature, were calculated.

The 3D observations of the inner skin structures revealed that the structures in the abdominal and eyelid tissues were fundamentally different. Several appendages, such as hair follicles and sweat glands, were observed in the eyelid skin but rarely in the abdominal skin. It was thought that because we used the eyelid skin excised from a region, 1–2 mm below the eyelashes, the hair follicles of the eyelashes were observed. In addition, they assumed to be eyelashes based on the thickness of the hair measured from the images. In the abdominal skin, oxytalan fibres, which are rich in fibrillin‐1, exhibiting a candelabra‐like structure, were observed just below the basement membrane, as previously reported.^1^, ^2^ However, in the eyelid skin, a complex entangled network of elastin fibres was observed below the basement membrane, which did not show the candelabra‐like structure. The image observation and analysis indicated that the proportion of fibrillin‐1 in the eyelid skin was higher than that in the abdomen skin. Although it was unclear whether these differences were due to congenital differences during tissue development or acquired differences due to ultraviolet rays and mechanical movements, it was interesting to understand the mechanism of elastin fibre formation.

In the aged skin from the eyelid and abdomen, the elastin fibres had a short, shrunk and spherical shape compared to the young skin. Because these alterations were common in the eyelid and abdominal skin, they might be because of the intrinsic and chronic physiological process of aging, ischemia and inflammation and might not depend on characteristic differences, such as on the effects of ultraviolet (UV) irradiation or mechanical irritation, between body parts. In contrast, although these altered parameters were common in the eyelid and abdominal skin, the degree of alteration with age was more significant in the eyelid skin. In addition, the number of fibre branches decreased with aging in the eyelid skin but not in the abdominal skin. It has been reported that the fibrous structure of the skin is associated with wrinkle formation and skin elasticity.^22^, ^23^ More dynamic alterations in the eyelid skin associated with aging may indicate that the eyelid skin is more fragile and prone to wrinkle formation and loss of elasticity than the abdominal skin. In addition, these differences in the fragility of fibre may be due to the differences in the frequency of photo‐exposure of the eyelids and abdomen. Truncation of fibrillin‐rich microfibrils in photo‐exposed skin, visualized in 2D, has been reported.^30^ The 3D observation and computational analysis performed in this study further support the previous data. Elastin‐degrading enzymes, UV‐induced reactive oxygen species and frequent mechanical movements might cause these changes in elastin fibres in the eyelid skin.^3^, ^4^ Alternatively, it might also be due to the differences in basic fibre properties, such as the elastin fibre network in the eyelid and the high content of fibrillin‐1, as mentioned above. However, some limitations should be noted. First, in this study, we used the skin samples of the abdomen and eyelids from donors belonging to different races. Individuals with different geographical ancestry have been reported to display structural and compositional differences in their skin.^31^ Therefore, these differences may be because of the use of skin samples from donors of different races. Second, we focused on skin samples from women donors in this study. Gender differences in the physiology and internal structure of skin have previously been reported^32^; therefore, further research is needed on skin from male donors.

In this study, we analysed elastin fibre structure in the young and aged eyelid and abdominal skin using 3D imaging and computational analysis. We show that 3D observation and structural analysis of the skin internal structure can quantitatively explain the degeneration of elastin fibres in more detail than conventional methods. Moreover, our computational 3D structural analysis would be effective not only for elastin fibres but also for correctly capturing the skin structure. It is expected that applying this 3D analysis method to skin tissues will contribute to a better understanding of age‐related changes in fibres and the development of novel wrinkle treatments.

## CONFLICT OF INTEREST

The authors declare no conflict of interest.

## Supporting information

Supplementary Material

Supplementary Material

## References

[1] Surber C , Brandt S , Cozzio A , Kottner J . Principles of skin care in the elderly. G Ital Dermatol Venereol. 2015;150:699–716. 26349927

[2] Doubal S , Klemera P . Visco‐elastic response of human skin and aging. J Am Aging Assoc. 2002;25:115–7. 23604906 10.1007/s11357-002-0009-9 PMC3455245

[3] Naylor EC , Watson RE , Sherratt MJ . Molecular aspects of skin ageing. Maturitas. 2011;69:249–56. 21612880 10.1016/j.maturitas.2011.04.011

[4] Böck P , Stockinger L . Light and electron microscopic identification of elastic, elaunin and oxytalan fibers in human tracheal and bronchial mucosa. Anat Embryol. 1984;170:145–53. 10.1007/BF00318999 6517349

[5] Cotta‐Pereira G , Guerra Rodrigo F , Bittencourt‐Sampaio S . Oxytalan, elaunin, and elastic fibers in the human skin. J Invest Dermatol. 1976;66:143–8. 1249442 10.1111/1523-1747.ep12481882

[6] Braverman IM , Fonferko E . Studies in cutaneous aging: I. The elastic fiber network. J Invest Dermatol. 1982;78:434–43. 7069221 10.1111/1523-1747.ep12507866

[7] Sherratt MJ . Tissue elasticity and the ageing elastic fibre. Age. 2009;31:305–25. 19588272 10.1007/s11357-009-9103-6 PMC2813052

[8] Kielty CM , Sherratt MJ , Shuttleworth CA . Elastic fibres. J Cell Sci. 2002;115:2817–28. 12082143 10.1242/jcs.115.14.2817

[9] Weihermann AC , Lorencini M , Brohem CA , de Carvalho CM . Elastin structure and its involvement in skin photoageing. Int J Cosmet Sci. 2017;39:241–7. 27731897 10.1111/ics.12372

[10] Kohl E , Steinbauer J , Landthaler M , Szeimies RM . Skin ageing. J Eur Acad Dermatol Venereol. 2011;25:873–84. 21261751 10.1111/j.1468-3083.2010.03963.x

[11] Kligman AM . Early destructive effect of sunlight on human skin. JAMA. 1969;210:2377–80. 5395389

[12] Friedman O . Changes associated with the aging face. Facial Plast Surg Clin North Am. 2005;13:371–80. 16085282 10.1016/j.fsc.2005.04.004

[13] Bonta M , Daina L , Muţiu G . The process of ageing reflected by histological changes in the skin. Rom J Morphol Embryol. 2013;54:797–804. 24322030

[14] Mora Huertas AC , Schmelzer CE , Hoehenwarter W , Heyroth F , Heinz A . Molecular‐level insights into aging processes of skin elastin. Biochimie. 2016;128–129:163–73. 10.1016/j.biochi.2016.08.010 27569260

[15] Schlotzer‐Schrehardt U , Stojkovic M , Hofmann‐Rummelt C , Cursiefen C , Kruse F , Holbach L . The pathogenesis of floppy eyelid syndrome: involvement of matrix metalloproteinases in elastic fiber degradation. Ophthalmology. 2005;112:694–704. 15808264 10.1016/j.ophtha.2004.11.031

[16] Alfonso‐Jaume MA , Bergman MR , Mahimkar R , Cheng S , Jin ZQ , Karliner JS , et al. Cardiac ischemia‐reperfusion injury induces matrix metalloproteinase‐2 expression through the AP‐1 components FosB and JunB. Am J Physiol Heart Circ Physiol. 2006;291:H1838–46. 16699069 10.1152/ajpheart.00026.2006

[17] Cheung PY , Sawicki G , Wozniak M , Wang W , Radomski MW , Schulz R . Matrix metalloproteinase‐2 contributes to ischemia‐reperfusion injury in the heart. Circulation. 2000;101:1833–9. 10769285 10.1161/01.cir.101.15.1833

[18] Prajapati RT , Eastwood M , Brown RA . Duration and orientation of mechanical loads determine fibroblast cyto‐mechanical activation: monitored by protease release. Wound Repair Regen. 2000;8:238–46. 10886814 10.1046/j.1524-475x.2000.00238.x

[19] Swartz MA , Tschumperlin DJ , Kamm RD , Drazen JM . Mechanical stress is communicated between different cell types to elicit matrix remodeling. Proc Natl Acad Sci U S A. 2001;98:6180–5. 11353845 10.1073/pnas.111133298 PMC33442

[20] Lindsey M , Wedin K , Brown MD , Keller C , Evans AJ , Smolen J , et al. Matrix‐dependent mechanism of neutrophil‐mediated release and activation of matrix metalloproteinase 9 in myocardial ischemia/reperfusion. Circulation. 2001;103:2181–7. 11331260 10.1161/01.cir.103.17.2181

[21] Debelle L , Tamburro AM . Elastin: molecular description and function. Int J Biochem Cell Biol. 1999;31:261–72. 10216959 10.1016/s1357-2725(98)00098-3

[22] Lee JY , Kim YK , Seo JY , Choi CW , Hwang JS , Lee BG , et al. Loss of elastic fibers causes skin wrinkles in sun‐damaged human skin. J Dermatol Sci. 2008;50:99–107. 18201872 10.1016/j.jdermsci.2007.11.010

[23] Langton AK , Graham HK , Griffiths CEM , Watson REB . Ageing significantly impacts the biomechanical function and structural composition of skin. Exp Dermatol. 2019;28:981–4. 31152614 10.1111/exd.13980 PMC6851988

[24] Kubota SI , Takahashi K , Nishida J , Morishita Y , Ehata S , Tainaka K , et al. Whole‐body profiling of cancer metastasis with single‐cell resolution. Cell Rep. 2017;20:236–50. 28683317 10.1016/j.celrep.2017.06.010

[25] Tainaka K , Kuno A , Kubota SI , Murakami T , Ueda HR . Chemical principles in tissue clearing and staining protocols for whole‐body cell profiling. Annu Rev Cell Dev Biol. 2016;32:713–41. 27298088 10.1146/annurev-cellbio-111315-125001

[26] Susaki EA , Ueda HR . Whole‐body and whole‐organ clearing and imaging techniques with single‐cell resolution: toward organism‐level systems biology in mammals. Cell Chem Biol. 2016;23:137–57. 26933741 10.1016/j.chembiol.2015.11.009

[27] Susaki EA , Tainaka K , Perrin D , Yukinaga H , Kuno A , Ueda HR . Advanced CUBIC protocols for whole‐brain and whole‐body clearing and imaging. Nat Protoc. 2015;10:1709–27. 26448360 10.1038/nprot.2015.085

[28] Tainaka K , Kubota SI , Suyama TQ , Susaki EA , Perrin D , Ukai‐Tadenuma M , et al. Whole‐body imaging with single‐cell resolution by tissue decolorization. Cell. 2014;159:911–24. 25417165 10.1016/j.cell.2014.10.034

[29] Susaki EA , Tainaka K , Perrin D , Kishino F , Tawara T , Watanabe TM , et al. Whole‐brain imaging with single‐cell resolution using chemical cocktails and computational analysis. Cell. 2014;157:726–39. 24746791 10.1016/j.cell.2014.03.042

[30] Watson RE , Griffiths CE , Craven NM , Shuttleworth CA , Kielty CM . Fibrillin‐rich microfibrils are reduced in photoaged skin. Distribution at the dermal‐epidermal junction. J Invest Dermatol. 1999;112:782–7. 10233772 10.1046/j.1523-1747.1999.00562.x

[31] Langton AK , Sherratt MJ , Sellers WI , Griffiths CE , Watson RE . Geographical ancestry is a key determinant of epidermal morphology and dermal composition. Br J Dermatol. 2014;171:274–82. 24484315 10.1111/bjd.12860

[32] Rahrovan S , Fanian F , Mehryan P , Humbert P , Firooz A . Male versus female skin: what dermatologists and cosmeticians should know. Int J Womens Dermatol. 2018;4:122–30. 30175213 10.1016/j.ijwd.2018.03.002 PMC6116811

